# Biscuits: a systematic review and meta-analysis of improving the nutritional quality and health benefits

**DOI:** 10.1186/s43014-021-00071-z

**Published:** 2021-10-01

**Authors:** Mahamadé Goubgou, Laurencia T. Songré-Ouattara, Fabrice Bationo, Hagrétou Lingani-Sawadogo, Yves Traoré, Aly Savadogo

**Affiliations:** 1 Doctoral School of Sciences and Technology, Laboratory of Applied Biochemistry and Immunology (LABIA), University Joseph KI-ZERBO, Ouagadougou, Burkina Faso; 2 Department of Food Technology (DTA), Research Institute of Applied Sciences and Technology (IRSAT), Ouagadougou, Burkina Faso

**Keywords:** Biscuit, Technology, Nutrition, Health benefits

## Abstract

Biscuits are ready-to-eat foods that are traditionally prepared mainly with wheat flour, fat, and sugar. Recently, biscuits’ technologies have been rapidly developed to improve their nutritional properties. This study aimed to determine the strategies of improving the nutritional quality of biscuits and the potential health benefits associated with them. A systematic review and meta-analysis were conducted, including articles on biscuits improved by technological processes and raw materials variation. Studies were searched from Google Scholar, PubMed, Scopus, and Web of Science published between 1997 and 2020, in English and French. The meta-analysis was performed using RStudio software, version 4.0.4 to classify the biscuits. One hundred and seven eligible articles were identified. Rice, pea, potato, sorghum, buckwheat, and flaxseed flours were respectively the most found substitutes to wheat flour. But the meta-analysis shown that the copra and foxtail millet biscuit fortified with amaranth, the wheat biscuits fortified with okra, and rice biscuits fortified with soybeans had a high protein content. These biscuits therefore have a potential to be used as complementary foods. The substitution of sugar and fat by several substitutes lead to a decrease in carbohydrates, fat, and energy value. It has also brought about an increase in other nutrients such as dietary fiber, proteins/amino acids, fatty acids, and phenolic compounds. Among the sugar and fat substitutes, stevia and inulin were respectively the most used. Regarding the use of biscuits in clinical trials, they were mainly used for addressing micronutrient deficiency and for weight loss.

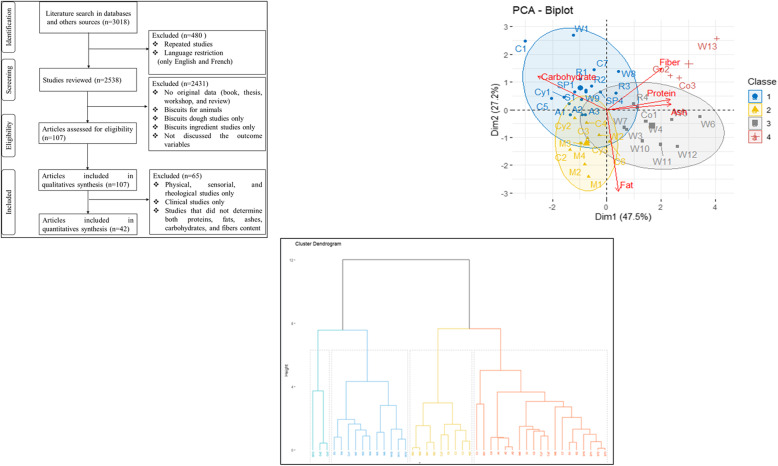

## Introduction

Many efforts have been made to develop food products that can improve people’s health (Coutinho de Moura et al. 2014; Galanakis 2020; Granato et al. 2010). In developing countries, while overweight/obesity is increasing in all age groups, undernutrition persists and coexists with obesity and the burden of diet-related diseases (World Health Organization 2021). Furthermore, recent outbreaks of infectious diseases, such as malaria, Ebola virus disease, HIV pandemic, and the recent COVID-19 crisis emphasize the need to adopt healthy diets (Galanakis 2020). As a result, many health organizations have been incentivized to the development and consumption of improved food, such as food enriched with functional constituents (French Ministry of Health 2006; Hercberg et al. 2008; World Health Organization 2015). For this purpose, several popular or traditional foods have been used as vehicles in fortification strategies.

Popular foods are effective vehicles for nutrient incorporation and are thus targeted by a growing and increasingly demanding market for the management of health disorders (Granato et al. 2010). Among these foods, biscuits show potential as improved food (Nogueira & Steel 2018) to meet nutritional needs or prevent diet-related illnesses. Biscuits offer several possibilities for the management of human nutrition-related disorders. They are widely consumed as snacks or as complement to other foods. They present varied forms and pleasant flavors, have long shelf lives, and provide convenience (Agama-Acevedo et al. 2012; Manley 2011). Therefore, the production and consumption of biscuits have considerably increased worldwide (Canalis et al. 2017). For products with the fast-moving consumer goods category, the biscuit market is among the leading ones (Apeda agri exchange 2020). The biscuit market reached $76.385 billion at the end of 2017 and expected to reach USD 121 billion by 2021 and USD 164 billion by 2024 at compound annual growth rate (CAGR) of 3.7 and 5.08%, respectively (Apeda agri exchange 2020). The highest per capita consumption of biscuits in the world is approximately 13 kg per year (Canalis et al. 2017). The wide consumption of biscuit makes it an ideal product for fortification (Kadam & Prabhasankar 2010). However, some biscuits are even used as part of nutritional strategies to tackle several chronic and nutrition-related diseases, such as nutrient deficiencies, diabetes, obesity, cardiovascular diseases, and cancers (Canalis et al. 2017; Singh & Kumar 2017; van Stuijvenberg et al. 2001). Innovations in biscuit technologies and recipes have resulted in a wide range of biscuit products, both the forms and nutritional properties (Denis 2011; Filipčev et al. 2014; Swapna & Jayaraj Rao 2016). Biscuits have many functional forms and can be enriched with mineral and vitamin complexes (MVC) or nutrient-rich complementary ingredients, and formulated for infants, children, the elderly and those with special needs such as the obese and diabetics (Davidson 2019). The fortification ability of biscuits and their high consumer acceptance have led to them receiving more attention for formulating functional foods or nutraceuticals. Several clinical trials have also been carried out on the efficacy of improved biscuits against illnesses and prevention of chronic and nutrition-related diseases (Kriengsinyos et al. 2015; Kekalih et al. 2019; Buffière et al. 2020). Compared to the large number of biscuits currently available, the number of research studies examining improved biscuits and their use in clinical trials is quite limited. Therefore, this paper reviews the different improved biscuits and their use in clinical trials against illnesses and risk of chronic diseases such as malnutrition, diabetes, hypertension, cardiovascular disease, cancer, obesity, low HDL cholesterol, high triglyceride level, etc.

## Methods

### Search strategy

**Fig. 1:**
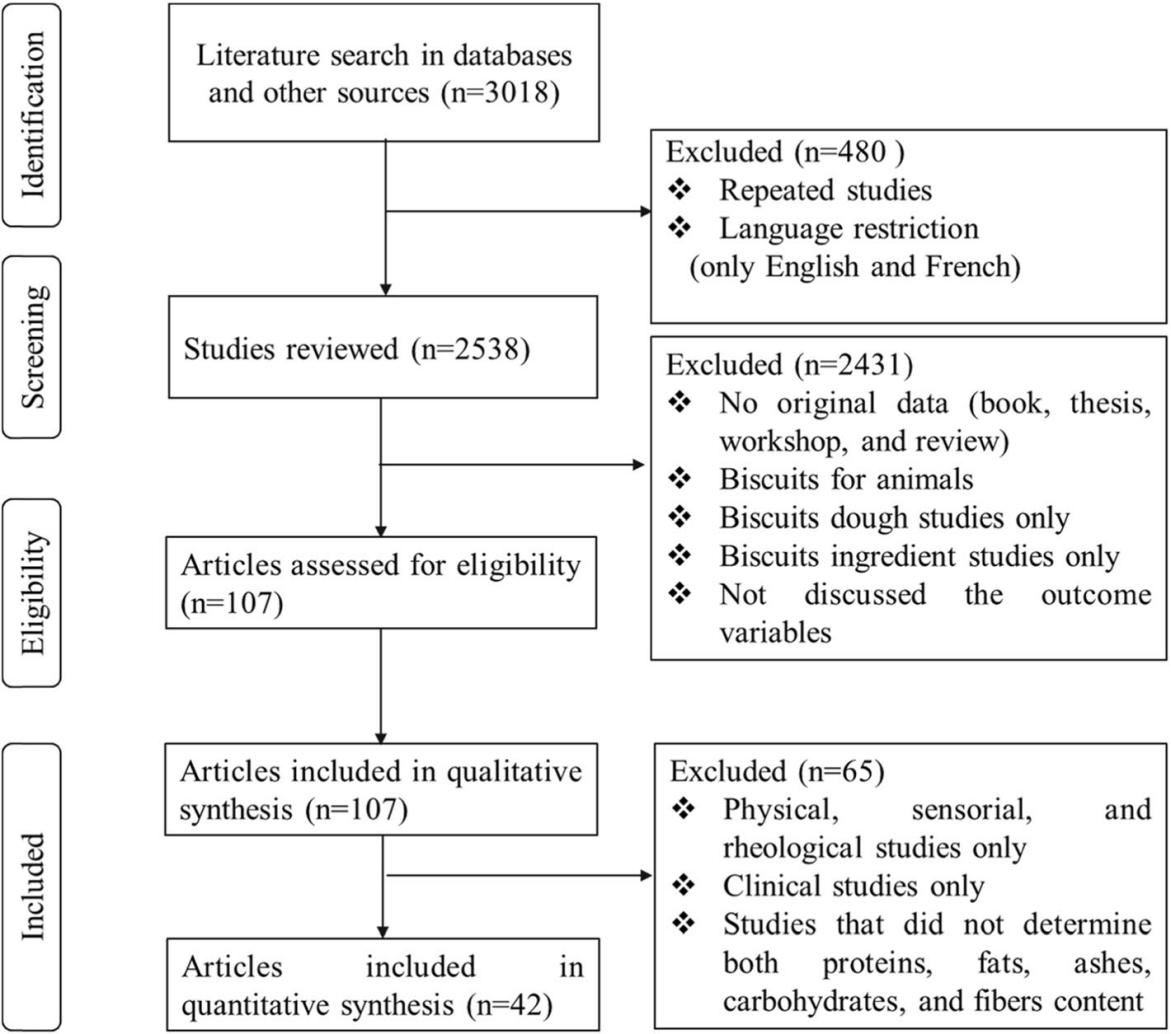
Flow chart of screening process

At first, this study searched in many databases as Google Scholar, PubMed, Scopus, Web of Science with keywords. These keywords are: Biscuit OR Cookie OR Cracker AND Improved quality, Biscuit OR Cookie OR Cracker AND High quality, Improved Biscuit OR Cookie OR Cracker, High quality Biscuit OR Cookie OR Cracker, Biscuit OR Cookie OR Cracker AND Health benefits, Biscuit OR Cookie OR Cracker AND Clinical trial, Biscuit OR Cookie OR Cracker AND Trial. Secondly, references of all identified articles were searched to get additional studies. Figure 1 shows the articles selection procedure and information used in this study.

### Inclusion criteria

Studies with the following criteria were eligible for inclusion: 1) studies that have investigated the effect of total or partial substitution of wheat flour; 2) studies that have investigated the effect of substitution or the reduction of sugar or fat for the production of biscuits; 3) studies that have investigated novelties in the processing of improved biscuits; 4) studies that have investigated the use of improved biscuits in clinical trials.

The term “biscuit” derives from the Latin word *biscoctus*, which means twice-cooked/baked (Chavan et al. 2016). The origin of biscuit dates back to Roman times to resolve food preservation (Chavan et al. 2016). In baking, the word “biscuit” includes several groups of products. It is called “biscuit” in the United Kingdom and in France, “cookie” and “cracker” in the United States, and “scone” in New Zealand (Chavan et al. 2016; Denis 2011). For other authors, biscuits are termed interchangeably with cookies in the United Kingdom and Asia (Cauvain & Young 2008). However, some differences can be noted between the products. Biscuits are generally made from short dough, which is undeveloped and lacks extensibility (Xu et al. 2020) while cookies are made from soft dough, which has high sugar, high fat, and low moisture content (Delcour & Hoseney 2010). Cracker, for instance, is traditionally made from soft dough and is a thin and crisp product baked from unsweetened and unleavened dough (Xu et al. 2020). In this paper, the term biscuit is used for biscuits, cookies, and cracker.

### Exclusion criteria

Letters, comments, communications, reviews, thesis, and animal studies were excluded. Also, excluded were articles published in languages other than English and French, citations with no abstracts and/or full texts, duplicate studies, and articles focusing solely on biscuits ingredients and dough studies. Furthermore, for the quantitative synthesis (meta-analysis), the articles reporting only on physical, sensorial, and rheological properties, clinical trials, and those that did not determine jointly proteins, fats, ashes, carbohydrates, and fibers contents were excluded.

### Synthesis of findings

The included articles were analysed qualitatively using a thematic analysis approach. Then, all articles were synthesized by systematic reading.

## Results

### Study characteristics

In our initial search, we found 3018 articles, of which 480 were identified as repeated studies or language restriction (only English and French) and were excluded. After reviewing titles and abstracts, 2431 articles were also excluded for the following reasons: 1) no original data (book, thesis, workshop, and review); 2) biscuits for animals; 3) biscuit ingredients studies only; and 4) biscuits dough studies only. After this, there were 107 articles left to include in the qualitative synthesis (Fig. 1).

For the quantitative synthesis (meta-analysis), 65 articles were excluded due to the following reasons: 1) physical, sensorial, and rheological studies only; 2) clinical trials studies only; and 3) studies that did not determine jointly proteins, fats, ashes, carbohydrates, and fibers contents. Finally, 42 articles were included (Fig. 1).

One hundred and seven articles were identified for the systematic review, from which sixty-nine were based on partial or total substitution of wheat flour by other flours (Table 1). From the sixty-nine substituted wheat flour biscuits, twenty-nine were based on total substitution of wheat flour. Besides, 20/107 and 11/107 dealt with, respectively, the high amount of sugar and fat in biscuits (Tables 1 and 2). Finally, 19 / 107 articles were based on the use of biscuits in clinical trials (Table 3).

**Table 1:** Improved materials used to substitute wheat flour in biscuit production

Product added	Improvement elements	Substitution level (%)	Reference
Buckwheat	Processing properties, sensory, and textural characteristics, protein content, and gluten-free biscuits	50–100	Sedej et al. 2011; Torbica et al. 2012; Hadnađev et al. 2013; Mancebo et al. 2015; Kaur et al. 2015
Sorghum	Dietary fiber and low calorie; Fat, protein, ash, and calorific values as compared to wheat biscuits	25–45, 50, and 100	Okpala & Okoli 2011; Banerjee et al. 2014; Rai et al. 2014; Songré-Ouattara et al. 2016, 2017
Maize	Gluten-free biscuits	100	Rai et al. 2014; Mancebo et al. 2015
Rice	Processing properties, sensory, and textural characteristics, gluten-free biscuits	50–100	Ceesay et al. 1997; Torbica et al. 2012; Hadnađev et al. 2013; Radočaj et al. 2014; Rai et al. 2014; Mancebo et al. 2015; Benkadri et al. 2018; Sulieman et al. 2019
Pearl millet	Fat, protein, ash, and calorific values as compared to wheat biscuits	100	Rai et al. 2014
Foxtail millet flour	Low phytates and tannins, increased polyphenols	9	Singh & Kumar 2017
Copra	Fiber and protein contents	51	Singh et al. Singh & Kumar 2017
Pea	Protein, fat, iron, and crude fiber contents	5–100	(Adeola & Ohizua 2018, Benkadri et al. 2018 Chinma et al. 2011, Dhankhar et al. 2019, Han et al. 2010, Okpala & Okoli 2011, Silky & Tiwari 2014, Zucco et al. 2011)
Bean	Physical and nutritional characteristics	25, 50, 75, and 100	Han et al. 2010; Zucco et al. 2011
Lentil	Physical and nutritional characteristics	100	Han et al. 2010; Zucco et al. 2011
Potato	Protein, fat, minerals, crude fiber, staling, flavor, ash, and sugar	10, 14, 16, 18–100	(Abou-Zaid & Elbandy 2014; Onabanjo & Ighere 2014; Adeyeye & Akingbala 2015; Songré-Ouattara et al. 2016; Adeola & Ohizua 2018; Sulieman et al. 2019)
Cheese	Digestible source of fat and protein and rich source of vitamin A, B2 and B12 and highly bioavailable minerals as calcium and zinc	30, 40, and 50	Swapna & Jayaraj Rao 2016
Date powder	Fiber	10, 20, 30, and 40	Dhankhar et al. 2019
Soy	Protein, anti-nutrients, amino acids and vitamins profile and sensory evaluation	5, 10, 15, and 20	Hu et al. 2013, Loo et al. 2017; Ghoshal & Kaushik 2020, Adeyeye 2020
Flaxseed	ω-3 (α-linolenic acid), dietary soluble and insoluble fibers and lignans	5, 12, 15, 30, 50, and 75	Hassan et al. 2012; Coutinho de Moura et al. 2014; Austria et al. 2016; Omran et al. 2016; Kuang et al. 2020
Almond	Bioactive components	–	Jung et al. 2018; Bowen et al. 2019; Pasqualone et al. 2020
Groundnut	Energy, protein, calcium, and iron	–	Ceesay et al. 1997
Fenugreek	Protein, lysine, dietary fiber, Ca, Fe, alkaloids, flavonoids, and saponins	5, 10, and 15	Hooda & Jood 2005
Mushrooms	Protein, fiber, ash, fat, potassium, phosphorus, magnesium, calcium, vitamin B3, vitamin C, texture, flavor, and sensory acceptability	5, 10, 15, and 20	Ayo et al. 2018; Biao et al. 2020
Acha	Carbohydrate	100	Ayo et al. 2018
Cocoyam	Carbohydrate	100	Okpala & Okoli 2011; Akujobi 2018
Tigernut flour	Protein, magnesium, iron, zinc, vitamin E, vitamin A, and folic acid	30, 50, 70, 80, 85, 90 and 95	Chinma et al. 2011; Akujobi 2018
Gums	Effect of gums addition on color, appearance and flavor and overall acceptability	100	Kaur et al. 2015
Multi-micronutrients (Iron, zinc, iodine, and vitamin A)	Iron, zinc, iodine, and vitamin A content	–	Nga et al. 2009, 2011; Songré-Ouattara et al. 2016
Amaranth flours	Amino acid profile and high bioavailability of protein	13, 20, and 40	De la Barca et al. 2010; Singh & Kumar 2017
Fish and crustacean	Lysine, amino acids	3, 5, and 6	Ibrahim 2009; Abou-Zaid & Elbandy 2014
Grape (skin and seeds)	Proteins, ash, lipids, carbohydrates, vitamins, and phenolic compounds (tannins, phenolic acids, anthocyanins, and resveratrol)	15	Karnopp et al. 2015; Kuchtová et al. 2016, 2018
Moringa	Carotenoid, protein, and dietary fiber	5, 10, and 15	Songré-Ouattara et al. 2016, 2017
Spirulina	Carotenoid	4, 6, and 8	Songré-Ouattara et al. 2016, 2017
Hemp flour	Protein, crude fibers, minerals, and essential fatty acids	10, 20, 30, and 40	Radočaj et al. 2014
Okra powder	Protein, ash, and fiber	5, 10, 15, 20, and 25	Akoja & Coker 2018
Tea leaves	Protein, fiber, minerals, and antioxidant properties	2.67, 2.02, 1.35, and 0.68	Radočaj et al. 2014
Mustard meal	Sensory quality maintained, nutritional and functional properties improved	5, 10, 15, 20, 25 and 30	Hassan et al. 2012
Barley meal	Sensory quality maintained, nutritional and functional properties improved	5, 10, 15, 20, 25 and 30	Hassan et al. 2012
Teff flour	Essential amino acids, iron, calcium, copper, zinc, aluminum, and barium	10, 20, 40, and 100	Coleman et al. 2013; Mancebo et al. 2015
Oat (flour and bran)	Influence of different packaging on the storage period, fibers content	100	Serial et al. 2016; Swapna & Jayaraj Rao 2016; Lee & Kang 2018; Duta et al. 2019
Inulin	Fibers content	–	Serial et al. 2016
Whey protein	Dietary fiber, protein, lipids, carbohydrate, sugar and energy	25	Aggarwal et al. 2016, Hassanzadeh-Rostami et al. 2020
*Agaricus bisporus* polysaccharide	Ash, protein, fat and total dietary fibers	3, 6, and 9	Sulieman et al. 2019
Banana (flour and peel)	Phenolic compound, starch digestibility, and glycemic index	5, 10, 15, 30, 45, and 50	Ovando-Martinez et al. 2009; Agama-Acevedo et al. 2012; Arun et al. 2015; Adeola & Ohizua 2018
Mango peel	Dietary fiber, polyphenols and carotenoids	5, 7.5, 10, 15, and 20	Ajila et al. 2008
Fruit and vegetable residue	Fiber and mineral	20, 25, and 35	Ferreira et al. 2013
Citrus (orange and lemon) by-products	Dietary fiber	0, 5, 10, and 15	Kohajdova et al. 2011
Grapefruits by-products	Dietary fiber	5, 10, and 15	Kohajdova et al. 2013
Watermelon rind powder	Dietary fiber, total phenolic content, glycemic index, and antioxidant activity	10, 20, and 30	Naknaen et al. 2016
Sour cherry pomace	Total polyphenols, total anthocyanins, and antioxidant activity	10 and 15	Šaponjac et al. 2016
Berries pomace	Linoleic acid and α -linolenic acid	20 and 30	Šarić et al. 2016
Pomegranate peel	Protein, dietary fiber, minerals, antioxidant activity, and β-carotene content	10	Srivastava et al. 2014
Apple pomace	Antioxidant properties, total dietary fiber, and minerals content	3, 6, 9, 15, and 20	Mir et al. 2015; Sudha et al. 2016
Olive pomace	Polyphenols and fiber	–	Conterno et al. 2019
Pomace of rowanberry, rosehip, blackcurrant, and elderberry	Dietary fiber, vitamins, and phenolic compounds	20	Tańska et al. 2016
Pumpkin	Dietary fiber	10 and 15	Turksoy & Ozkaya 2011
Carrot pomace	Dietary fiber	10 and 15	Turksoy & Ozkaya 2011
Carob by-products (germ and seed peel)	Protein, fiber and antioxidant activity	Carob peel: 0–9; Carob germ: 0–18	Martin-Diana et al. 2016
Beet molasses	Biscuit spread, fracturability, and storability	10–50	Filipčev et al. 2014, 2015
Glucomannan and xanthan	Fiber content	–	Jenkins et al. 2008
Plant Stanol Ester	Phytosterols	–	Kriengsinyos et al. 2015
Fructo-oligosaccharides	Fiber content	20	Tuohy et al. 2001
Gum (guar and xanthan)	Technological properties, fiber content	11	Tuohy et al. 2001; Benkadri et al. 2018

**Table 2:** Sugar substitutes and their potential effect on biscuit quality

Sugar substitute	Nature	Sugar rate (%)	Results	References
Maltitol and FOS-Sucralose		100		Aggarwal et al. 2016
Raftilose	Oligofructose	20	Reduce sugar content	Gallagher et al. 2003
Stevia	*S. rebaudiana* leaves	0.06–0.08-0.1-0.14, 25, 50, 75, and 100	High fiber content, angiotensin-converting enzyme and α-amylase inhibitory activity, and antioxidant effect	Vatankhah et al. 2015; Pourmohammadi et al. 2017; Góngora Salazar et al. 2018
Erythritol	Sweetener	25, 50, 75, and 100	Partial replacement of sucrose with up to 50% erythritol had sensory and physical quality characteristics comparable with cookies prepared with 100% sucrose	Lin et al. 2010
Arabinoxylan oligosaccharides	Complex carbohydrates	30	Reduction of sucrose and increase of fiber levels	Pareyt et al. 2011
Isomalt	Polyol	3, 6, 9, and 12	Reduction of sucrose	Pourmohammadi et al. 2017
Maltodextrin	Starch	2.5–5–7.5-10	Reduction of sucrose	Pourmohammadi et al. 2017
Isomalt, maltodextrin, stevia	–	6–2.5-0.06	Biscuits were more comparable to one elaborate with sucrose, and with the highest acceptance level in sensory evaluations	Pourmohammadi et al. 2017

**Table 3:** Shortening substitutes and their potential effect on biscuit quality

Fat substitute	Nature	Substitution rate (%)	Results	References
Inulin	Non-digestible dietary fiber	20 and 25	Textural and sensory properties maintained; Dietary fiber increased, weakened lubrication of biscuit dough, reduction of energy density	Rodríguez-García et al. 2013; Błońska et al. 2014; Banerjee et al. 2014; Krystyjan et al. 2015; Onacik-Gür et al. 2015; Canalis et al. 2017
Rice starch	Complex carbohydrates	20	Native and modified rice starch effective	Lee & Puligundla 2016
Corn fiber	Complex carbohydrates	30	Fat reduction and fiber fortification	Forker et al. 2012
Lupine extract	Complex carbohydrates	30	Fat reduction and fiber fortification	Forker et al. 2012
Wheat bran fibers	Plant fibers	10, 20, and 30	Texture of biscuits was greatly dependent on the texture of the dough	Erinc et al. 2018
Candelilla wax-canola oil oleogels	Oil	30–40	Decrease of saturated fatty acids (63.4% → 32.3%)	Jang et al. 2015
Puree of canned green peas		75	Sensory assessment	Romanchik-Cerpovicz et al. 2018
Lecithin		3	Lecithin (3%, sunflower based) achieved similar sensory quality as fat biscuit	Onacik-Gür et al. 2015
Maltodextrin	Complex carbohydrates	50, 60, and 70	Texture maintained, low-fat biscuit	Sudha et al. 2007; Chugh et al. 2013
Guar gum	Complex carbohydrates	–	Low-fat biscuit	Chugh et al. 2013
Polydextrose	Complex carbohydrates	50, 60, and 70	Texture maintained	Sudha et al. 2007, Aggarwal et al. 2016
Red palm oil	Oil	–	β-carotene	van Stuijvenberg et al. 2001
Goat fat	Oil	100	Functional and nutritional properties increased	Costa et al. 2019
Flax oil	Oil	100	ω-3 fatty acids	Hassan et al. 2012
Sunflower oil	Oil	100	Free of *trans* and low-saturated fats	Tarancon et al. 2013; Onacik-Gür et al. 2015
Olive oil	Oil	100	Free of trans and low-saturated fats	Tarancón et al. Tarancon et al. 2013

### Synthesis of improvement studies

A listing of materials used to improve the nutritional quality of biscuits is summarized in Table 1. They includes, mushroom (Biao et al. 2020; Jung & Joo 2010), banana flour (Ovando-Martinez et al. 2009), pigeon pea flour (Adeola & Ohizua 2018; Silky & Tiwari 2014), sweet potato (Adeola & Ohizua 2018; Adeyeye & Akingbala 2015; Onabanjo & Ighere 2014), cheese (Swapna & Jayaraj Rao 2016), tigernut (Chinma et al. 2011), flaxeed (Hassan et al. 2012), fenugreek seeds (Hooda & Jood 2005), grape (Karnopp et al. 2015; Kuchtová et al. 2016, 2018), fish and crustacean (Ibrahim 2009; Abou-Zaid and Elbandy 2014), hemp flour (Radočaj et al. 2014), and decaffeinated green tea leaves (Radočaj et al. 2014). In addition, several by-products such as germ, and peel (Martin-Diana et al. 2016) have been used in partial substitution of cereal flour in the formula of biscuit.

Among the 69 articles based on the substitution of flour that are included in this study, rice (Benkadri et al. 2018; Ceesay et al. 1997; Hadnađev et al 2013; Mancebo et al. 2015; Radočaj et al. 2014; Rai et al. 2014; Sulieman et al. 2019; Torbica et al. 2012), pea (Han et al. 2010; Chinma et al. 2011; Okpala & Okoli 2011; Zucco et al. 2011; Silky & Tiwari 2014; Adeola & Ohizua 2018; Benkadri et al. 2018; Dhankhar et al. 2019, potato (Abou-Zaid & Elbandy 2014; Onabanjo & Ighere 2014; Adeyeye & Akingbala 2015; Songré-Ouattara et al. 2016; Adeola & Ohizua 2018; Sulieman et al. 2019), sorghum (Banerjee et al. 2014; Okpala & Okoli 2011; Rai et al. 2014; Songré-Ouattara et al. 2016, 2017), buckwheat (Hadnađev et al. 2013; Kaur et al. 2015; Mancebo et al. 2015; Sedej et al. 2011; Torbica et al. 2012), and flaxseed (Hassan et al. 2012; Coutinho de Moura et al. 2014; Austria et al. 2016; Kuang et al. 2020; Omran et al. 2016) have received more attention (Table 1).

### Quantitative synthesis

**Fig. 2:**
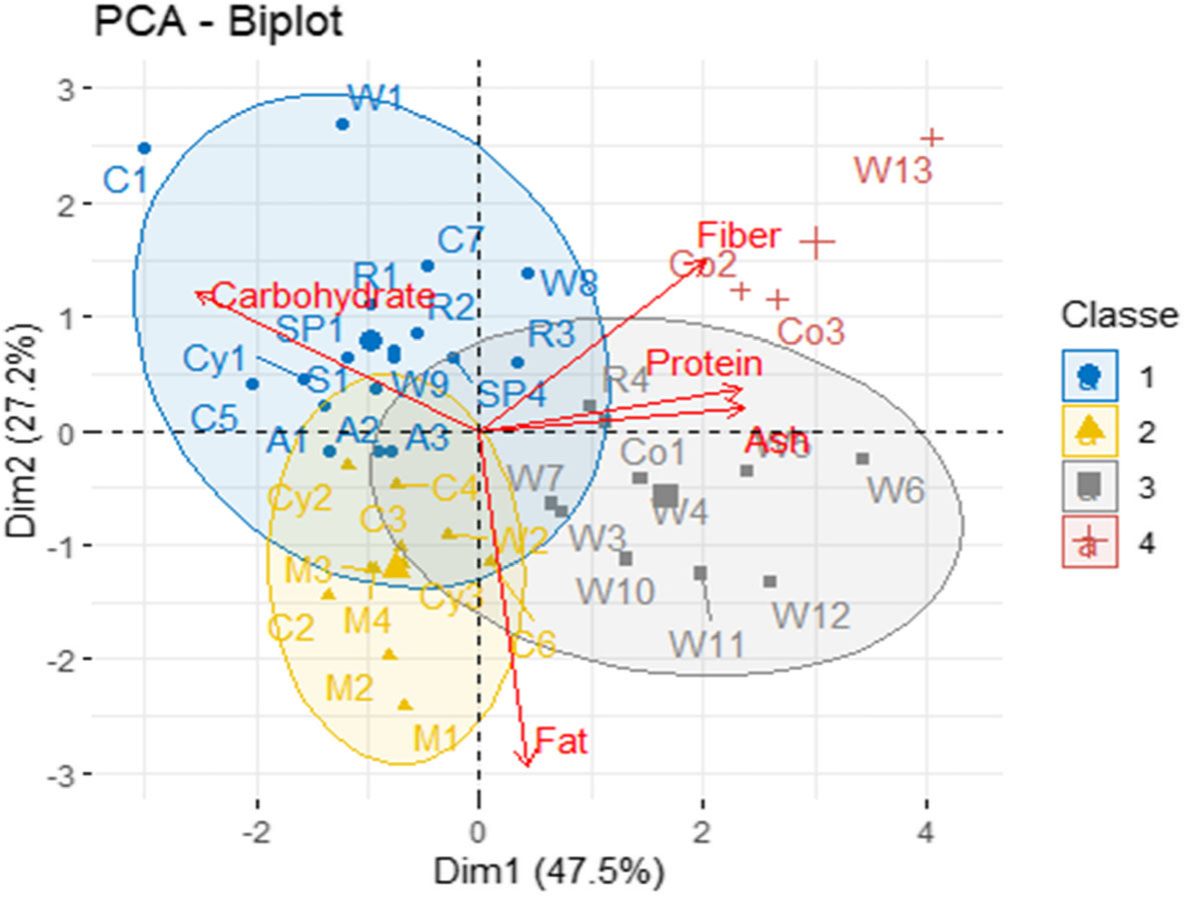
PCA biplot of the proximate composition to 42 biscuits

The Principal Component Analysis (PCA) performed with FactoMineR and FactoExtra package of the RStudio software, version 4.1.4 on the proximate composition of 42 biscuits (Fig. 2) showed a spread of individual trees along the main axes, which explained 74.7% of the total variation, with 47.5% of variation associated to dimension 1 and 27.2% to dimension 2. The dispersion along dimension 1 was mainly related to ash, protein, carbohydrate, and crude fiber; while the dispersion along dimension 2 was mainly linked to variation in fat content. The PCA biplot gives four classes of biscuits. Wheat biscuits (C1, C5, C7), wheat fortified biscuits (W1, W8, W9), all sweet potato biscuits (SP1-SP4), aya biscuits (A1-A3), sorghum biscuit (S1), rice biscuits (R1-R3), cocoyam biscuits (Cy1) seemed to be more similar between to one another for high carbohydrate content, less fiber, less protein, and less ash content, as revealed by the PCA biplot (Fig. 2). Also, wheat biscuits (C2, C3, and C6), wheat fortified biscuits (W2), all maize and maize fortified biscuits (M1-M4), and cocoyam biscuits (Cy2 and Cy3) seemed to be more similar to one another for high fat content. The third class of biscuit included wheat fortified biscuits (W3-W7, and W10-W12), copra and foxtail millet biscuits (Co1), and rice fortified biscuits (R4) with high content of protein, ashes and fiber. The PCA analysis revealed a last group of three biscuits (W13, Co2 and Co3) that seemed to be more similar to one another for high content of fiber.

**Fig. 3:**
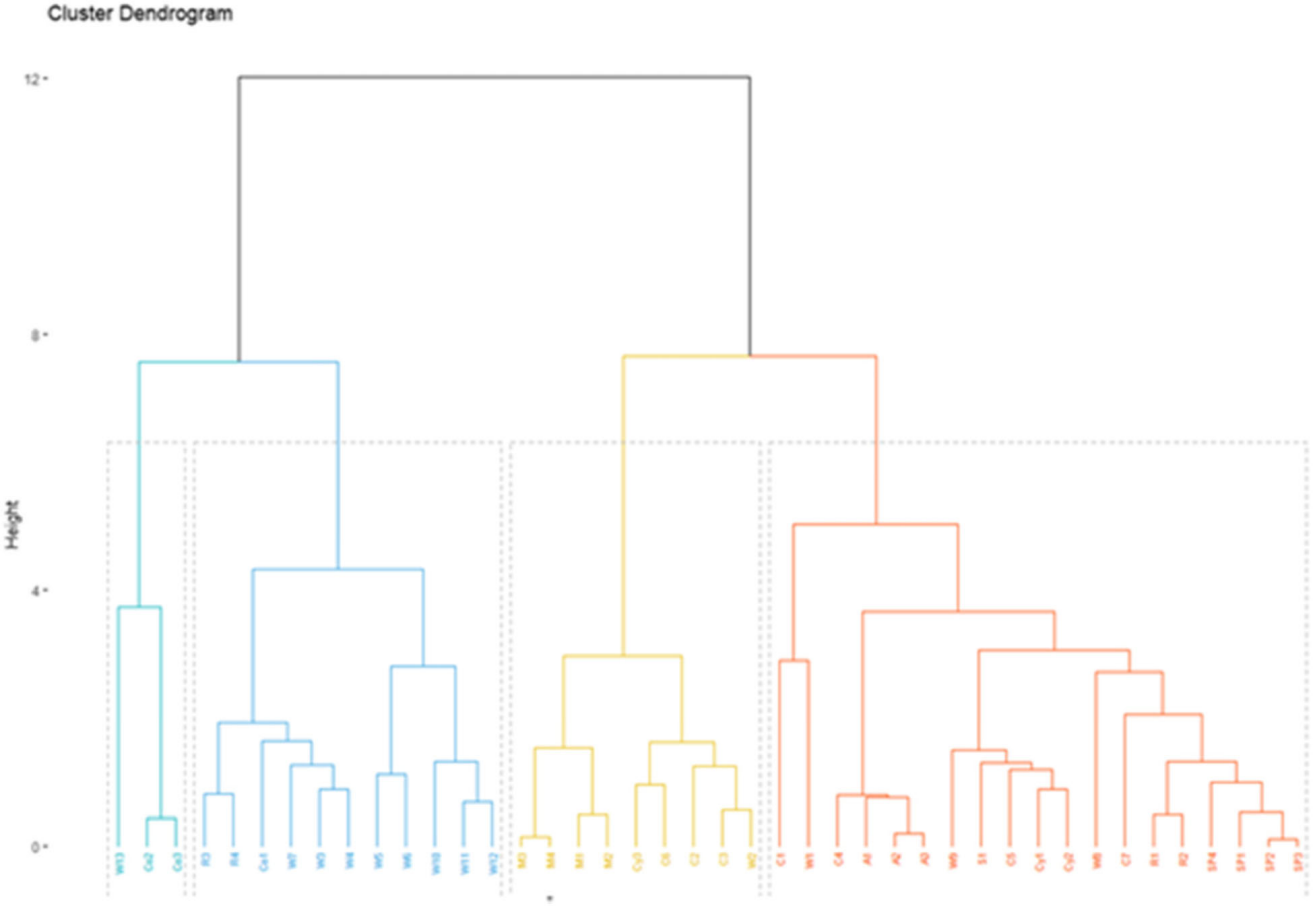
Hierarchical ascending classification of proximate composition of 42 biscuits

The Hierarchical Ascending Classification (HAC) or dendrogram performed with FactoMineR and FactoExtra package of the RStudio software, version 4.1.4 on the proximate composition of the 42 biscuits is shown in Fig. 3 and gives four classes of biscuits. The first group consisted of biscuits W13, Co2 and Co3. They contain the highest fiber content. The second group is represented by the biscuits R3, R4, Co1, W7, W3, W4, W5, W6, W10, W11, and W12 which revealed the highest proteins contents. The third group counted M3, M4, M1, M2, Cy3, C6, C2, C3, and W2 and presented the highest lipids contents. At last, the fourth group was composed of biscuits C1, W1, C4, A1, A2, A3, W9, S1, C5, Cy1, Cy2, W8, C7, R1, R2, SP4, SP1, SP2, and SP3 and were characterized by the highest carbohydrates contents.

All of the biscuits included in the PCA and HAC analyses had a good acceptability (Dhankhar et al. 2019; Omran et al. 2016; Singh & Kumar 2017).

The code associated with each point is the unique identifier of each biscuit: C = control biscuits, with wheat as the only flour constituent (Akoja & Coker 2018; Ayo et al. 2018; Banerjee et al. 2014; Coutinho de Moura et al. 2014; Dhankhar et al. 2019; Hu et al. 2013; Omran et al. 2016), W = wheat based biscuits, with wheat as the majority of the flour and with additional improvement constituent (Akoja & Coker 2018; Coutinho de Moura et al. 2014; Dhankhar et al. 2019; Omran et al. 2016), S=Sorghum or sorghum based biscuits (Banerjee et al. 2014; Songré-Ouattara et al. 2017), M = maize or maize based biscuits (Costa et al. 2019), SP = sweet potato or sweet potato based biscuits (Sulieman et al. 2019), Co = copra and foxtail millet or their based biscuits (Singh & Kumar 2017), R = rice or rice based biscuits (Adeyeye 2020), Cy = cocoyam or cocoyam based biscuits (Akujobi 2018), A = acha or acha based biscuits (Ayo et al. 2018). The different circles represent the three populations studied. Vectors show the relative weight of the variables moisture, ashes, lipids, proteins and carbohydrates, which determines the spread of points (individual trees) on the biplot.

### Biscuit and potential health benefits

Several studies have been carried out on the potential health benefits of biscuits for humans. These studies focused on the reduction or the substitution of sugar (Table 2) and / or shortening (Table 3) in biscuits production, the use of gluten-free flour as a substitute of wheat flour (Table 1), and the use of biscuits in clinical trials to combat or prevent some diseases (Table 4).

**Table 4:** The use of biscuit in clinical trial

Health concern	Additional product	Additional product properties	Administration mode	Result	References
Micronutrient deficiency	Multi-Micronutrient	Improvement of micronutrient status	30 g biscuits, 5 days/week /4 months	Improvement of the concentrations of hemoglobin (+ 1.87 g/L), plasma ferritin (+ 7.5 mg/L), body iron (+ 0.56 mg/kg body weight), plasma zinc (+ 0.61 mmol/L), plasma retinol (+ 0.041 mmol/L), and urinary iodine (+ 22.49 mmol/L); reduction of the risk of anemia (40%) and deficiencies of zinc (40%) and iodine (40%).	Nga et al. 2009
		Iron fortification	2 or 3 biscuits / 6 d/week/ 28 weeks	Improved iron status and reduction of blood lead concentrations (4.3 μg/dL to 2.9 μ g/dL for NaFeEDTA)	Bouhouch et al. 2016
	Roasted almonds	Monounsaturated fatty acids, polyunsaturated fatty acids, fiber, and vitamin E	56 g of almonds biscuits / day / 4 weeks	Decreased total cholesterol (5.5%), low-density lipoprotein cholesterol (4.6%), and non- high-density lipoprotein cholesterol (6.4%) and increased plasma α-tocopherol (8.5%) compared to the biscuit control.	Jung et al. 2018
Weight loss	Whey protein and wheat bran	Highest satiety feeling	50 g / day / 8 weeks	Control appetite (Composite appetite score: − 3.12), more decrease of energy intake (− 1531.13 KJ/day), body weight (− 2.91 Kg), waist circumference (− 4.44 cm), and serum insulin (− 2.31 mIU/L); more increase GLP-1 (+ 0.05) and more attenuate reduction of HDL-C level (+ 1.18 mg/dl) comparatively to control biscuits.	Hassanzadeh-Rostami et al. 2020
	Soy fiber	Rich source of dietary fiber	100 g/day / 12 weeks	Significant decrease of body weight (− 1.39 kg), body mass index (− 0.51), waist circumference (− 1.75 cm), diastolic blood pressure (− 3.82 mmHg), serum levels of total cholesterol (− 0.58 mmol/L), LDL-C (− 0.41 mmol/L), and glucose (− 0.95 mmol/L), body fat (− 0.71 kg), and trunk fat (− 0.64 kg) for those who consumed the supplemented biscuits comparatively to those who consumed the control biscuit.	Hu et al. 2013
	Flaxseed flour	Rich source of dietary fiber	100 g of biscuits / day / 60 days	Decrease body weight (− 0.83) and lower triacylglycerol levels (− 0.04 mmol/L) comparatively to control group	Kuang et al. 2020
Type 2 diabetes	Carbohydrate	Rich source of bioactive components	56 g /day / 8 weeks	Significant reduction of serum total cholesterol/HDL-C ratio in women those consumed almond snack compared to those who consumed biscuit snack (− 0.36 mmol/L vs. -0.14 mmol/L)	Bowen et al. 2019
	Glucomannan and xanthan	High fiber content	10 g / biscuit	Reduction of the glycemic index by 74% in healthy participants and by 63% in participants with diabetes	Jenkins et al. 2008
Post-prandial folate bioavailability	Folic acid	Folate plasma availability	_	Biscuit and custard have presented comparable folate bioavailability	Buffière et al. 2020
Serum cholesterol reduction efficacy	Plant stanol ester	Low-density lipoprotein cholesterol -(LDL-C-) lowering efficacy	1 biscuit / day / 2 weeks	Compared to the control, the total cholesterol, LDL-C, and the LDL/HDL ratio had serum reductions of 4.9, 6.1, and 4.3%, respectively	Kriengsinyos et al. 2015
Birth weight and perinatal mortality	groundnut	Reduce the retardation of fetal growth	2 biscuit (4.3 MJ*2)/day / 20 weeks	Increased weight gain in pregnancy (136 g) over the whole year and significantly increased birth weight (11.1% of babies with low birth weight for the intervention group against (17% for the control group).	Ceesay et al. 1997
Parasitic infections	Multi-Micronutrient	Decreased parasite load and improved cognitive outcomes	30 g biscuits, 5 days/week / 4 months	Decrease of *Ascaris* (− 2328 eggs per gram of feces) and Hookworm (− 156 eggs per gram of feces) and improve cognitive outcomes. These values are higher than those of the group consumed placebo (− 1200 for *Ascaris* and − 144 for Hookworm).	Nga et al. 2011
Gastrointestinal symptoms and autism spectrum disorders	Gluten-free biscuit (GFB)	Reduction of the prevalence of gastrointestinal symptoms and autism spectrum disorders behaviors	Gluten-free diet / 6 weeks	Significant (*P* < 0.05) decrease of the prevalence of gastrointestinal symptoms after intake of GFB (40.57% vs. 17.10%) against an insignificant increase in the regular diet group (RD) (42.45% vs. 44.05%). GFB also induces a significant decrease in behavioral disorders (80.03 vs. 75.82) against an insignificant increase in the regular diet group (79.92vs. 80.92).	Ghalichi et al. 2016
Prebiotics effect	Partially hydrolysed guar gum and fructo-oligosaccharides	Prebiotic effects	37.5 g / day / 21 days	Bifidobacterial numbers increased from pretreatment levels of 9. 10 log _10_ cells/g faeces and placebo levels of 9. 18 log_10_ cells/g faeces, to 9. 59 log_10_ cells/g faeces after ingestion of the experimental biscuits.	Tuohy et al. 2001
Neurocognitive outcomes	Soy protein	Protein dietary supplementation	Biscuits 5 days / week / 18 months	Improvements of nonverbal cognitive (fluid intelligence) performance for children who received soy protein than those who received ASFs. For example, beery visual-motor integration for children who received soy protein is 7.44 and 6.70 for children who received beef.	Loo et al. 2017

Considering the well-known deleterious consequences of sugar and fat in biscuits, there are more and more, several nutritive products that can replace sugar and fat in biscuits formulation with effect to decrease the calories, keep the sucrose and fat functionalities, and improve the nutritional values (dietary fiber, bioactive compounds, and minerals) (Tables 2 and 3).

For sugar substitutes, the nutritive sweeteners include maltitol, FOS-sucralose, isomalt, erythritol, arabinoxylan oligosaccharides, maltodextrin, and stevia (Gallagher et al. 2003; Lin et al. 2010; Pareyt et al. 2011; Vatankhah et al. 2015; Aggarwal et al. Aggarwal et al. 2016; Pourmohammadi et al. 2017; Góngora Salazar et al. 2018). Stevia is the most used as sugar substitute (Vatankhah et al. 2015; Pourmohammadi et al. 2017; Góngora Salazar et al. 2018).

Several materials have been used during biscuit processing for fat substitution and include inulin (Banerjee et al. 2014; Błońska et al. 2014; Canalis et al. 2017; Krystyjan et al. 2015; Onacik-Gür et al. 2015; Rodríguez-García et al. 2013), rice starch (Lee & Puligundla 2016), corn fiber (Forker et al. 2012), polydextrose (Aggarwal et al. 2016; Sudha et al. 2007), sunflower oil (Onacik-Gür et al. 2015; Tarancon et al. 2013), lecithin (Onacik-Gür et al. 2015), puree of canned green peas (Romanchik-Cerpovicz et al. 2018), candelilla wax–canola oil oleogels (Jang et al. 2015), maltodextrin (Chugh et al. 2013; Sudha et al. 2007), guar gum (Chugh et al. 2013), lupine extract (Forker et al. 2012), wheat bran fibers (Erinc et al. 2018), red palm oil (van Stuijvenberg et al. 2001), goat fat (Costa et al. 2019), flax oil (Hassan et al. 2012), and olive oil (Tarancón et al. Tarancon et al. 2013). Among them, inulin (Banerjee et al. 2014; Błońska et al. 2014; Canalis et al. 2017; Krystyjan et al. 2015; Onacik-Gür et al. 2015; Rodríguez-García et al. 2013), polydextrose (Aggarwal et al. 2016; Sudha et al. 2007), maltodextrin (Chugh et al. 2013; Sudha et al., 2007), and sunflower oil (Onacik-Gür et al. 2015; Tarancon et al. 2013) are the most used.

The use of biscuits in clinical trials has focused on the fight against some diseases but also on the evaluating of the effects of other foods ingredients on human health. It includes micronutrient deficiency (Nga et al. 2009, Bouhouch et al. 2016, Jung et al. 2018), weight loss (Hassanzadeh-Rostami et al. 2020; Hu et al. 2013; Kuang et al. 2020), type 2 diabetes (Bowen et al. 2019; Jenkins et al. 2008), post-prandial folate bioavailability (Buffière et al. 2020), serum cholesterol reduction efficacy (Kriengsinyos et al. 2015), birth weight and perinatal mortality (Ceesay et al. 1997), parasitic infections (Nga et al. 2011), gastrointestinal symptoms and autism spectrum disorders (Ghalichi et al. 2016), prebiotics effect (Tuohy et al. 2001), and neurocognitive outcomes (Loo et al. 2017) (Table 4). Biscuit has been used as a vehicle for specified nutrients, such as vitamins and minerals.

## Discussion

### Improving the nutritional quality of biscuits

The improvement in biscuit quality involves primarily novel recipes, process improvement, nutritional enrichment, and health promotion, as summarized in the following headlines.

#### Total and partial substitution of wheat flour

Wheat is the main source of flour used to produce biscuits (Chavan et al. 1993; Denis 2011). However, the nutritional quality of wheat flour is known to be limited (Chavan et al. 1993). Indeed, several bioactive components are unevenly distributed in wheat grains (Table 1). For example, around 50 to 60% of the minerals and vitamins are distributed in the bran, aleurone, and germ (Chavan et al. 1993). Consequently, these components are partially or totally removed during milling, leading to lower nutritional quality biscuits (Chavan et al. 1993). Therefore, it is proposed to use whole-grain flour to preserve the nutrients from the bran. Nonetheless, wheat grains still have protein quality inferior to most cereals (Chavan et al. 1993). Protein from wheat flour is a poor source of lysine, methionine, and threonine (Chavan et al. 1993). Refined wheat flour has more reduced nutritional quality with very low protein quality (Chavan et al. 1993). Another factor to be considered as limiting the nutritional quality of wheat flour is the presence of gluten. The high content of gluten in wheat flour has been a concern in public health due to gluten sensitivity, allergies, and coeliac disease (Mancebo et al. 2015; Rosell et al. 2014).

Given the concern with wheat flour’s nutritional quality, alternative flours have been explored (Benkadri et al. 2018; Chung et al. 2014; Mancebo et al. 2015). Wheat flour has been partially or totally substituted. Also, the sweet potato, the amaranth, the buckwheat, the pea and the acha showed to be interesting substitutes for wheat in biscuit production (Han et al. 2010; De la Barca et al. 2010; Hadnađev et al. 2013; Mancebo et al. 2015; Kaur et al. 2015; Adeyeye & Akingbala 2015; Ayo et al. 2018; Adeola & Ohizua 2018). Moreover, the flours’ protein quality has been improved using for example, fish proteins, whey, single-cell proteins, mushroom leaf protein isolates, legumes, and oilseeds (Abou-Zaid & Elbandy 2014; Biao et al. 2020; Jang et al. 2015). Interestingly, food waste or by-product (e.g., peels, germs, biomass waste such as leaves) are recovered and used as functional ingredients for the formulation of biscuit flour. Subsequently, xanthan gum, guar gum, arabic gum, agarose, β -glucan, and carboxymethyl cellulose (CMC) have been used to overcome technological aspects (i.e., rheological properties) attributed to gluten (Hadnađev et al. 2013; Songré-Ouattara et al., 2017; Xu et al. 2020).

The partial substitution of cereal flour with specific components has also been used to improve the nutritional quality and the potential health benefits of biscuits (Adeola & Ohizua 2018; Biao et al. 2020).

#### Novelties in the processing of improved biscuits

The improvement of the nutritional values of biscuits can be attributed to several innovations in the processing (Chung et al. 2014; Mancebo et al. 2015). The first challenge is associated with the production of the flour base. The particle size of flour is well known to influence the quality of biscuits (Mancebo et al. 2015). The incorporation of fine flours increases the biscuit hardness and decreases its spread. Besides, the coarse flours impact the biscuit textural (i.e., cohesiveness, spread) and organoleptic properties (i.e., mouthfeels) (Zucco et al. 2011). Subsequently, the flour processing is adapted, with compromise, according to the technological and nutritional aspects. However, refined flour of cereal used in biscuit processing is known to have lower nutritional quality and protein content than the whole cereal grain flour (Mancebo et al. 2015). Furthermore, roasting, precooking, defatting, germination, non-refining, and fermentation have been applied to prepare the ingredients and have also been showed to create novel flavors and improve the nutritional properties of the final biscuits (Mancebo et al. 2015; Omran et al. 2016; Singh & Kumar 2017; Sulieman et al. 2019).

Fermentation is a good way of incorporating enrichment ingredient that increases the nutritional quality of biscuits (Sulieman et al. 2019). For instance, using of 6% of fermented *Agaricus bisporus* polysaccharide flours in sweet potato and rice biscuits allowed to improve the biscuits’ nutritional and functional properties (Sulieman et al. 2019). However, the unfermented *Agaricus bisporus* polysaccharide flours have to be incorporated at a lower percentage (3%) to have good acceptability (Sulieman et al. 2019).

On the other hand, the germination of foxtail millet for the production of biscuit resulted in a decrease of anti-nutrients including phytates and tannins (Singh & Kumar 2017). It induced an increase of polyphenols content in biscuits (Singh & Kumar 2017). The use of germinated brown rice by partial or complete replacement of wheat flour in the production of biscuit has also led to the improvement of the nutritional quality of the biscuits (Chung et al. 2014).

Defatting has also been used to improve the acceptability of biscuits. This was the case with biscuits produced using defatted flaxseed flour. They were more appreciated than the biscuits produced with whole fat flaxseed flour (Omran et al. 2016).

#### Nutritional, and sensory quality of improved biscuit

The association of various cereals and the use of constituents with nutritional and technological interests have improved the nutritional, sensory, and functional properties of biscuits. A short overview of articles that mentioned the nutritional, physico-chemical and sensory quality of biscuits shows that the content of several functional nutrients such as protein, fiber, ω-3 fatty acids, dietary fibers, antioxidants, vitamins, and mineral has been enhanced (Costa et al. 2019; Swapna & Jayaraj Rao 2016). For example, the biscuits produced with oats and cheese had high nutritive value with 12.53 and 12.89% protein, 2.70 and 2.75% minerals and 0.62 and 0.60% beta-glucan (Swapna & Jayaraj Rao, 2016). Whole wheat combined with sorghum has been used to produce biscuits with good acceptability (ranking 7 on hedonic scale of 9 point) (Banerjee et al. 2014). The acceptability of biscuits can increase with the rate of sorghum ranging from 35 to 40% (Banerjee et al. 2014). Ghoshal and Kaushik (2020) have produced high protein biscuits, adding defatted soy flour up to 20% in the formulation without affecting their overall acceptability (Ghoshal & Kaushik 2020).

Regarding gluten-free biscuits, it is shown that the non-wheat flour sources significantly influenced the overall acceptability, the weight, the moisture and the water activity of the biscuits (Benkadri et al. 2018; Mancebo et al. 2015). Gluten-free flour biscuits have lower spread and greater hardness than wheat flour based-biscuits (Mancebo et al. 2015). The effect of the gums used to compensate the rule of gluten in gluten-free biscuit processing, was investigated by Kaur et al. (2015), who concluded that the qualities of buckwheat biscuits with xanthan gum were comparable to those made with wheat flour (Kaur et al. 2015).

In the case of the use of wholegrain and the substitution of wheat flour, the studies of Sedej et al. (2011) have shown no significant difference from sensory evaluation between the whole buckwheat grain biscuits and whole-wheat biscuits (Sedej et al. 2011).

The use of by-product (e.g., pomace and peel) generally increases the nutritional quality of biscuit, specially the content of protein, fiber, minerals, essential fatty acids and antioxidant potential (Martin-Diana et al. 2016). For the physical property, by-products reduce the pasting viscosity and the lightness of the biscuit; but increase the pasting temperature of the biscuit dough (Mir et al. 2015).

**Fig. 4:**
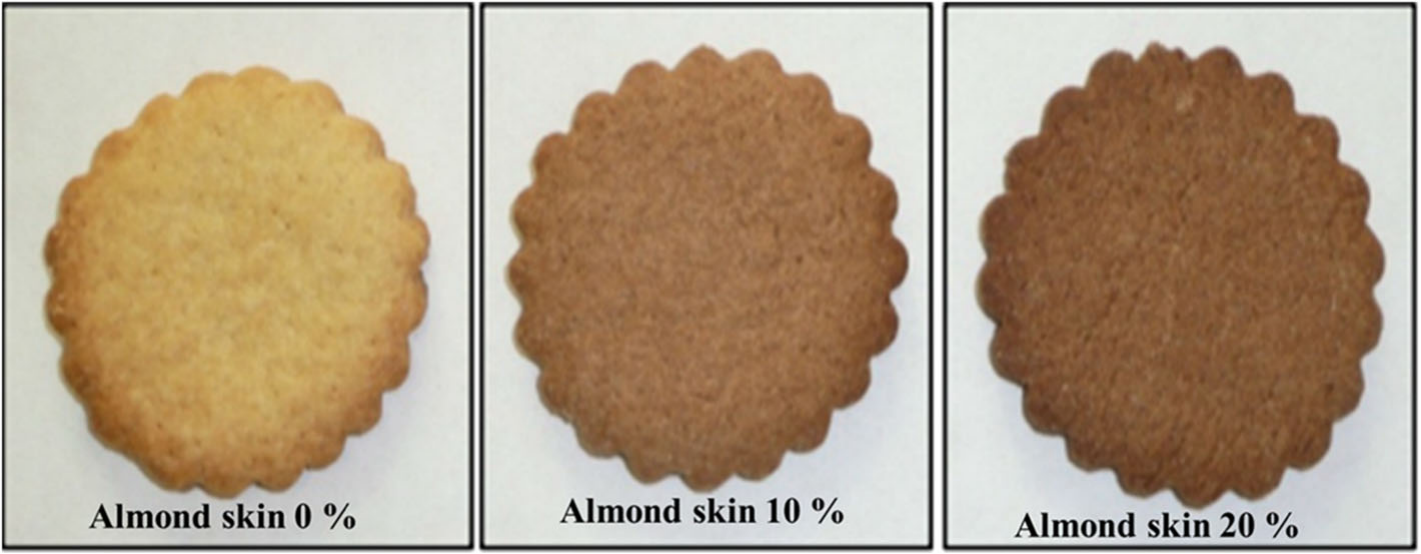
Biscuits enriched with almond skins showing the change of color

The use of improved materials has presented best proximate composition but had lower acceptability (Coutinho de Moura et al. 2014; Songré-Ouattara et al. 2016) due to the change of different sensory parameter such as color, taste, texture, aroma, odor (Fig. 4) (Pasqualone et al. 2020; Šarić et al. 2016; Tańska et al. 2016).

*From left to right: Control biscuit prepared with 100% wheat flour; and biscuits prepared by adding 10 and 20% of almond skin powder in the level of wheat flour (*Pasqualone et al. 2020*)*.

The PCA analysis showed two groups (3 and 4) of biscuits with a good potential for children’s diets due to the high protein content and average carbohydrate level. These groups are characterized by biscuits produced with copra and foxtail millet (Co1, Co2 and Co3) blended with amaranth (Singh & Kumar 2017) and wheat biscuit supplemented with soy fiber (W13) (Hu et al. 2013). The PCA analysis also showed that biscuits produced with 100 and 95% wheat flour (C1, C2, C3, and W1) have high carbohydrate and fat contents.

### Biscuit and potential health benefits

#### Control of biscuits’ sugar content

Sugars are the second major constituent of biscuit (Chavan et al. 2016; Sahin et al. 2019; van der Smam & Renzetti 2019). Biscuits contain a high amount of sugar (10–30%), which influences their techno-functional properties (e.g. taste and flavor) and increases their shelf-life (Chavan et al. 2016; Serial et al. 2016; van der Smam & Renzetti 2019). The sugar content significantly influences the organoleptic characteristics of biscuits. Depending on the temperature, the sugar content is responsible for the desired brown color (Perego et al. 2007). The crumbly and crispy textures are due to the undissolved sugar crystals and sugar recrystallization (Pareyt et al. 2009).

The high content of sugar in biscuits makes them high energy density foods, with an energy density of around 5 cal / g (Banerjee et al. 2014), which is over the recommended energy density for complementary food (4–4,25 cal / g) (CODEX CAC/GL 08 1991). However, for adults, this high energy density food has a concern in public health, because this is often linked with risks of some diseases such as cardiovascular diseases, diabetes, and obesity (Pourmohammadi et al. 2017; van der Smam & Renzetti 2019). This public health concern has led many health organizations to encourage the production of biscuits with reduced calories (French Ministry of Health Health 2006; Hercberg et al. 2008; World Health Organization 2015). For example, the WHO guideline recommends reducing the daily intake of free-sugars to 10% (World Health Organization 2015). In addition, the UK government has recommended bakery products with reduced calories of 20% (Sahin et al. 2019). Because of worldwide public-health campaigns claiming for no added sugar, biscuits with decreased calories are more and more common in the market (Aggarwal et al. 2016; Biguzzi et al. 2014; Denis 2011).

The decrease of sugar in biscuits formulas tends to alter the texture, sensory and hedonic properties (Biguzzi et al. 2014; Pareyt et al. 2009). A sugar reduction of up to 25% was linked to a significant of sweetness (Perego et al. 2007). Nevertheless, studies carried out in European countries, mainly France, have identified a significant decrease in the sugar content. This decrease varies between 2 to 15 g / 100 g in biscuits and cakes (Denis 2011). The studies of Dhankhar et al. (2019) showed that the reduction of sugar level in biscuits could be made up to 60% by using date powder as a sweetening agent for replacing sugar (Dhankhar et al. 2019).

#### Control of biscuits’ fat content

Fat is also one of the major constituents of biscuits. This constituent plays a substantial role in the nutritional quality of biscuits by increasing their tenderness and regulating their texture. Fat used in biscuits processing must have specific physicochemical and technological properties such as high melting point and plasticity (Costa et al. 2019; Tarancon et al. 2013). Fat is well-known to regulate the mechanical and rheological properties of biscuits (Colla et al. 2018). This constituent provides a good smell and taste to biscuits and greatly influences their convenient form and sensory properties. Fat can be from animal (butter, butter oil) or plant (palm oil, peanut oil, etc.) sources (Chavan et al. 2016; Pareyt et al. 2009).

Like sugar, the high fat content in biscuits can make them high energy density foods that are greatly recommended for children as complementary food. However, the high fat content constitutes a concern in public health, because it is often linked with risks of some diseases such as cardiovascular diseases, diabetes, cancer and obesity (Lee & Puligundla 2016; Okumura et al. 2017). From this point of view many health organizations have also issued recommendations for reducing fat content in food for adults. Besides, people with risks of cardiovascular diseases, diabetes, and obesity are looking to maintain a healthy diet including nutritious biscuits with no fat, low fat, and reduced fat (Colla et al. 2018; Erinc et al. 2018; French Ministry of Health 2006; Hercberg et al. 2008).

Given the above and considering the well-known deleterious consequences of the consumption of biscuits with high fat content, biscuits with fat reduced or without fat (Banerjee et al. 2014; Erinc et al. 2018) which maintain good sensory properties are required in the market. For this purpose, several ingredients have been used as fat substitutes during the biscuit processing (Table 3).

Some examples of fat substitutes include inulin, spreads and milk, b-glucan and amylodextrins, pectin, polydextrose, acetylated rice starch, high-oleic sunflower oil and inulin/ β-glucan/lecithin, puree of canned green peas, candelilla wax–canola oil oleogels, corn fiber, maltodextrin, guar gum and lupine extractare (Forker et al. 2012; Chugh et al. 2013; Banerjee et al. 2014; Krystyjan et al. 2015; Jang et al. 2015; Onacik-Gür et al. 2015; Lee & Puligundla 2016; Erinc et al. 2018; Romanchik-Cerpovicz et al. 2018).

#### Gluten-free biscuits

The high content of gluten in wheat flour has been a concern in public health due to food allergies, celiac disease and gluten sensitivity (Mancebo et al. 2015; Rosell et al. 2014). Therefore, wheat flour is substituted with several other flours to improve biscuits’ quality or prevent gluten-associated health disorders (Table 1). Some gluten-free cereals used in biscuit processing are rice (*O. sativa*), sorghum (*S. vulgare*), maize (*Z. mays*), and several minor grains such as the millets, especially pearl millet (*P. glaucum*), teff (*E. tef*), oat (*A. sativa*) (Adeyeye & Akingbala 2015; Coleman et al. 2013; Duta & Culetu, 2015; Mancebo et al. 2015; Rai et al., 2014; Songré-Ouattara et al. 2016; Torbica et al. 2012) (Table 1). Because of the lack of gluten, these products could be well tolerated in celiac disease patients as part of a gluten-free diet.

#### Use of improved biscuits in clinical trials

The objective of the use of improved biscuits in clinical trials is both to investigate the contribution of improved biscuits to the recommended nutrients intake of young children and the influence of the food matrix on the bioavailability of biscuit nutrients during digestion (Table 4) (Austria et al. 2016; Bowen et al. 2019; Buffière et al. 2020; Hu et al. 2013; Jenkins et al. 2008; Kriengsinyos et al. 2015).

Food that is rich in dietary fiber has been suggested to contribute to body weight loss, and lower triacylglycerol levels. Several studies have investigated the effect of supplemented biscuit with high fiber product on body weight, body composition, and blood lipids in overweight and obese subjects (Hassanzadeh-Rostami et al. 2020; Hu et al. 2013; Kuang et al. 2020). For example, the consumption of biscuits supplemented with soy fiber by overweight and obese college adults at breakfast for 12 weeks (approximately 100 g/day) has led to a loss of body weight, body mass index, and serum LDL-cholesterol concentrations compared to a control group, which received not supplemented biscuits (Hu et al., 2013). Kuang et al. (2020) have observed similar results with biscuits supplemented with flaxseed meal (Kuang et al. 2020).

The consumption of fortified biscuit with whey protein and wheat bran by overweight or obese people in a randomized controlled clinical trial during 8 weeks resulted in a loss of appetite, energy intake, and body weight, contrary to the overweight or obese who consumed not fortified biscuits (Hassanzadeh-Rostami et al. 2020).

Fortification of food with micronutrient is a strategy used in food programs to overcome micronutrient deficiency. Thus, biscuits are mostly used in clinical trials as vehicles for micronutrients, with the purpose to improve micronutrient status (Bouhouch et al. 2016; Nga et al. 2009). This food-based strategy is well-known to decrease the risk of anemia and deficiencies of micronutrients such as zinc and iodine. Bouhouch et al. (2016), when using the biscuit as a food vehicle fortified with ferrous sulfate (FeSO 4) and ferric sodium EDTA (NaFeEDTA) in a randomized controlled trial, showed an improved iron status of children (Bouhouch et al. 2016). Nga et al. (2009) obtained a decrease in the risk of anemia and deficiencies of zinc and iodine by 40% (Nga et al. 2009).

The consumption of multi-micronutrient fortified biscuits showed significant improvement in cognitive test results (Nga et al. 2009). Soy dietary protein used in the supplementation of biscuit showed greater improvement in nonverbal cognitive (fluid intelligence) performance compared with peers who received isocaloric beef or wheat biscuits (Loo et al. 2017).

The daily supplementation of pregnant women’s diet with high energy groundnut biscuits (4.3 MJ / day) during 20 weeks significantly increases the weight gain in pregnancy and the birth weight (Ceesay et al. 1997).

Ginger has been used in the production of biscuit for its good effect against nausea and vomiting during pregnancy. Thus, Basirat et al. (2009), when using ginger biscuits their clinical trial, showed that these food products have a positive effect on the remission of nausea during pregnancy (Basirat et al. 2009).

It has been reported that the consumption of multi-micronutrient fortified biscuits reduces the prevalence of parasitic infections compared to children who received unfortified biscuits (Nga et al. 2009, 2011).

The consumption of biscuits enriched with olive pomace led to a significant increase in the metabolic output of the gut microbiota (Conterno et al. 2019).

Autism spectrum disorder affects multiple systems of the body. It is the case for metabolic, gastrointestinal, immunological, mitochondrial, and neurological systems. Ghalichi et al. (2016) used a gluten-free diet (gluten-free pasta and biscuits and gluten-free breads) in a randomized clinical trial to determine their effect on gastrointestinal symptoms and autism spectrum disorders. They concluded that these gluten-free biscuits might be effective product to manage of the gastrointestinal symptoms and autism spectrum disorders behaviors (Ghalichi et al. 2016).

The studies of Clifton and Keogh concluded that wheat wholegrain biscuits have a lowering effect on cholesterol rate in the blood (Clifton & Keogh 2018). This is an advantage because the use of wheat wholegrain biscuit at breakfast is a convenient, easy and nutritious way to achieve 2 g /day of plant sterol intake, and its form lends itself to excellent daily compliance (Clifton & Keogh 2018).

## Conclusion

This review emphasizes the scientific information about the nutritional attributes of biscuits and their correlation with human health. The biscuit industry intends to improve human health through the development of a wide variety of biscuits in the form of food high in essential and/or functional nutrient. Biscuit products appear to be an excellent food vehicle matrix for the inclusion of a variety of innovative and healthy ingredients and offer various positive functional attributes. The physical, chemical, functional, and rheological properties of these products are significantly influenced by the raw material used and the production process. The functionality of biscuits is the combined result of their physical, chemical, functional, and rheological properties. The different treatments of biscuits leading to the improvement of the nutritional contents have been mainly evidenced as an increase in protein, fiber, and bioactive compounds and a decrease in the hydrolysis index, fat and sugar content. There are many future possibilities for the biscuits industries to develop a wider range of tailor-made functional food products. It is difficult to explain the correlation between biscuits consumption and disease prevention because this aspect is not completely understood. The clinical study is an important step towards improving the understanding of the influence of biscuits on human health. This aspect is not fully understood. The use of biscuits in clinical trials has shown good prospects in improving the diet by providing bioactive compounds such as essential fatty acids, proteins, dietary fibers, soluble polysaccharides, phenolic compounds, vitamins (A, C, F and E), and minerals (P, Mg, K, Na, Fe, Cu, Mn and Zn). However, it is noteworthy that these values need to be tested in vivo to confirm the physiological benefits on human health of biscuits consumption. Several clinical trials have been conducted with biscuits. But, further clinical trials with more participants and over a longer duration could provide a better understanding of the health benefits of improved biscuits. Also, the clinical trials are minimal compared to the numbers of improved biscuits that have been developed.

## Data Availability

Data and materials used include all the original reviewed articles which are available.
